# Prognostic value of T regulatory cells and immune checkpoints expression in tumor-draining lymph nodes for oral squamous cell carcinoma

**DOI:** 10.3389/fimmu.2024.1455426

**Published:** 2024-10-15

**Authors:** Krzysztof Piersiala, Eric Hjalmarsson, Vilma Lagebro, Pedro Farrajota Neves da Silva, Rusana Bark, Alexandra Elliot, Linda Marklund, Gregori Margolin, Susanna Kumlien Georén, Lars-Olaf Cardell

**Affiliations:** ^1^ Division of ENT Diseases, Department of Clinical Sciences, Intervention and Technology, Karolinska Institutet, Stockholm, Sweden; ^2^ Department of Otorhinolaryngology, Karolinska University Hospital, Stockholm, Sweden; ^3^ Department of Pathology and Cytology, Karolinska University Hospital, Stockholm, Sweden; ^4^ Medical Unit Head Neck, Lung and Skin Cancer, Karolinska University Hospital, Stockholm, Sweden; ^5^ Department of Surgical Sciences, Section of Otolaryngology and Head and Neck Surgery, Uppsala University, Uppsala, Sweden

**Keywords:** oral squamous cell carcinoma, tumor-draining lymph nodes, immune checkpoints, prognostic indicators, neoadjuvant immunotherapy

## Abstract

Despite the employment of extensive therapeutic strategies, OSCC recurrence and mortality rates persist at high levels. This underscores the shortcomings of current prognostic models and the urgency for refined biomarkers. This study explores the prognostic significance of tumor-draining lymph nodes (TDLNs) in OSCC, with a special focus on the quantification of T regulatory cells (Tregs) and the expression of immune checkpoints on T cells.

**Methods:**

Forty-nine OSCC patients were enrolled. One TDLN per patient was analysed using flow cytometry to profile immune-checkpoint expression (PD-1, CTLA-4, TIGIT, TIM-3, LAG-3) and other markers such as CD69, CXCR5 on CD4+, CD8+, and Tregs. Disease-free survival (DFS) and overall survival (OS) were assessed.

**Results:**

According to multivariate analysis, elevated levels of FoxP3+CD4+ and TIGIT+CD8+ cells in TDLNs correlated with significantly worse DFS, while high CXCR5+CD4+ levels were associated with better DFS. Notably, the expression of immune checkpoints on T cells within TDLNs showed significant associations with recurrence status. Patients experiencing recurrence exhibited heightened levels of T regulatory cells, CD4+PD-1+ and CD4+CTLA-4+, cells in TDLNs. Survival multivariate analyses revealed that T status emerged as an independent predictor of OS.

**Conclusion:**

The findings highlight the critical role of TDLNs in the immune microenvironment of OSCC and establish immune checkpoint expression on T cells as promising prognostic biomarkers. These insights upgrade the prognostic framework for OSCC and pave the way for individualized therapeutic strategies. The prognostic significance of TDLNs and a high expression of immune checkpoint inhibitors is a compelling argument for the adoption of neoadjuvant immunotherapy.

## Introduction

Oral squamous cell carcinoma (OSCC) is a form of cancer that develops in the squamous cells lining of the oral cavity, encompassing areas such as the mobile tongue, cheeks, floor of the mouth, gingiva in the upper and lower jaw, and the hard palate. Data from the Global Cancer Observatory (GCO) reveals that in 2020, there were 377,713 reported cases of OSCC worldwide. Alarmingly, projections indicate a potential 40% increase in the incidence of OSCC by the year 2040 (1). The standard of care (SOC) for locally advanced OSCC involves surgical resection followed by adjuvant radiation/chemoradiation or definite chemoradiotherapy alone. Despite this aggressive approach, patients face unfavourable overall survival, with a five-year overall survival (OS) of approximately 50-60%. Additionally, the extensive treatment often leads to a decreased post-treatment quality of life, including swallowing or speech problems (2).

OSCC is notably characterized by its tendency to exhibit subclinical nodal metastasis, even in the early stages of the disease. The presence of metastasis in regional lymph nodes significantly worsens the prognosis (3). Due to the high incidence of nodal metastases, it is a standard practice to treat the neck in patients diagnosed with OSCC. For advanced tumors, therapeutic ipsilateral neck dissection (TND) followed by radiotherapy or chemoradiotherapy is widely accepted as the SOC. In cases of early-stage disease, elective neck dissection (END) or sentinel node biopsy is commonly employed. Despite this extensive treatment approach, the recurrence rate in patients with OSCC is high and ranges from 20-40% (4–6). The majority of patients relapse within 18 months (7) and recurrence is one of the major causes of low survival rates of OSCC after definitive therapy. The absence of reliable predictors, especially for individuals in the early stages of the disease, presents a major challenge in identifying those at risk of recurrence.

Recently, the use of novel therapeutics, such as cancer immunotherapeutics has gained increased attention. Immune checkpoint inhibitors (ICIs) have revolutionized oncological treatments and significantly improved survival for several types of cancer. Unfortunately, head- and neck cancer has so far not been part of this success, since only few patients show a lasting positive response to the treatment (8). Recently, the neoadjuvant setting for immunotherapy received attention for the treatment of head and neck squamous cell carcinoma (HNSCC) (9–12). Neoadjuvant therapy has become an increasingly promising approach as it allows for early treatment of micrometastatic disease, de-escalation of the therapy, and can increase the chances of a successful outcome. As mentioned above, the standard surgical protocol in oral cancer includes the excessive removal of lymph nodes, especially those in the proximity of the tumour. This in turn might be one of the main causes for the relatively poor effect of ICIs when used in an adjuvant therapy. In animals, neoadjuvant immunotherapy was shown to induce changes in the TDLNs that include increasing numbers and activity of T cells as well as DCs and decreasing numbers of regulatory T cells (13, 14). These changes can result in a more robust and effective immune response against the cancer cells, leading to better treatment outcomes.

Nevertheless, despite the acknowledged pivotal role of TDLNs in orchestrating immunotherapeutic responses and modulating anti-cancer immunity in animal models, scant attention has been directed toward TDLNs in human studies. In this study, we conducted a multiparameter flow cytometric analysis on TDLNs obtained from 49 with OSCC. The primary objective was to elucidate the expression profiles of immune checkpoints PD-1, CTLA-4, TIGIT, TIM-3, LAG-3, as well as CD69 and CXCR5 on CD4+, CD8+, and regulatory T cells. Furthermore, we sought to discern the prognostic relevance of the aforementioned expression patterns on T cells within TDLNs concerning Disease-Free Survival (DFS) and Overall Survival (OS).

## Materials and methods

### Patients and samples

Prospective enrollment for this study included patients who met specific inclusion criteria: 1) a diagnosis of primary OSCC, 2) tumor excision combined with a sentinel node-assisted elective neck dissection or sentinel node biopsy alone performed at Karolinska University Hospital, Stockholm, Sweden, between March 2019 and June 2022, and 3) a willingness to participate in the study. In brief, sentinel node (TDLNs) identification is conducted using SPECT-CT imaging, and its location is intraoperatively confirmed through gamma probe detection and the injection of indocyanine green (ICG), followed by visualization with near-infrared light. For detailed information on the sentinel node procedure at Karolinska University Hospital, please consult the paper by Kågedal et al. (15) Exclusion criteria were as follows: 1) systematic autoimmune diseases, 2) synchronous or previous second malignancies or hemo-lymphopoietic malignancies, and 3) any other acute or chronic condition that could influence the immunological environment in the lymph nodes. None of the patients received neoadjuvant chemotherapy or any other cancer treatment prior to inclusion in the study.

Following the collection of sentinel nodes, the fresh samples were immediately transferred to the Pathology Department at Karolinska University Hospital, where a designated pathologist separated parts of the TDLNs for analysis at the laboratory which were placed in pre-chilled MACS Tissue Storage Solution (Cat# 130-100-008, Miltenyi Biotec) on ice and utilized within 1 hour for subsequent analysis.

For the dissociation of specimens, the Tumour Dissociation KIT (Miltenyi Biotec #130-100-008) was used, involving both mechanical and enzymatic processes. Post-dissociation, the cells underwent filtration through a 100µm cell strainer (BD biosciences #352360). Subsequently, the cells were re-suspended in brilliant stain buffer (BD biosciences #563794) at a concentration of 40*10^6 cells/ml for downstream analysis. Finally, the samples underwent cryopreservation and were stored at -180 degrees Celsius till further analysis.

OSCC patients usually have 1-5 TDLNs identified in sentinel node procedure. For this project, only one TDLN per patient was analyzed. In patients who had a metastasis in a TDLN, this TDLN was used for analysis. If more than two nodes were metastasis positive, the node localized closest to the tumour was chosen (neck level 1> level 2> level 3). For metastasis negative node, the TDLN localized nearest the tumor was chosen (neck level 1> level 2> level 3).

### Sample preparation

Cryopreserved single-cell suspensions from selected patients were thawed and at first live/dead staining with LIVE/DEAD Fixable Aqua Dead Cell Stain Kit (Cat no L34957, Thermo Fisher Scientific Inc) was performed according to the manufacturer’s recommendations. After a wash, cells were incubated with Human Fc-block (BD Biosciences) and then with the following antibodies in the dark for 25 minutes at 4 Celsius degrees: anti-CD3 (BUV395), anti-CD4 (BUV496), anti-CD8 (BV510), PD-1 (BB700), CTLA-4 (BV421), TIM-3 (BB515), LAG-3 (AF-700), anti-CXCR5 (BV605), anti-CD69 (BUV805), anti-CD25 (BV711), anti-CD127 (PE). Staining was followed by two washing steps performed with PBS, 400g, for 5 minutes. For FoxP3 intracellular staining, cells were fixed and permeabilized using BD Cytofix/Cytoperm™ solution (BD Biosciences, 560098) according to the manufacturer’s protocol. For washing steps, Perm Wash Buffer (BD Biosciences, 554723). Cells were stained with anti-FoxP3+ (PE-CF594) antibody. After two additional washes, cells were resuspended in PBS with 1% paraformaldehyde (HistoLab #02178) and analyzed on LSR FORTESSA (BD Biosciences). Analysis of the flow cytometry data was performed with FlowJo version 10.8.0 (LLC, USA).

### Follow-up

All enrolled patients underwent regular follow-ups. Monitoring occurred with three-month intervals during the initial two years after surgery, and with six-month intervals from the third to the fifth year. Disease-free survival (DFS) was defined as the duration from surgery to the onset of recurrence, death from any cause, or the latest follow-up. Overall survival (OS) was defined as the period from surgery to the date of cancer-related death or the most recent follow-up.

### Statistics

Statistical analyses were performed with GraphPad Prism version 10.1.2 (GraphPad Software, La Jolla, CA, USA). Differences in expression of markers between subgroups were calculated by multiple t-test in followed by Holm–Sidak correction. Continuous variables were reported as mean ± standard deviation (SD). Univariate correlations were performed using Pearson correlation coefficients. P < 0.05 (*) was considered significant, and P < 0.01 (**), P < 0.001 (***), P < 0.0001 (****) were considered highly significant. Survival analysis was performed using SPSS software (version 29.0.1.0; SPSS Inc., Chicago, IL, USA). The survival curves were developed using the Kaplan-Meier method, the log-rank test was used to compare survival distributions of individual index levels. Univariate and multivariate Cox proportional hazards regression models were accessed to analyze the associations between the expression of analyzed markers and survival outcomes.

### Ethical approval

All procedures conducted in studies involving human participants adhered to the ethical standards set by the institutional and/or national research committee and complied with the principles outlined in the 1964 Helsinki Declaration and its subsequent amendments or equivalent ethical standards. Informed consent was obtained from all individual participants included in the study. The study received approvals from the Regional Ethics Committees under the reference numbers 2015/1650-31/2 and 2019-03518.

## Results

### Patients’ characteristics

Forty-nine consecutive patients who met the inclusion criteria were included in the study. Among them, 30 were males (61.2%), and 19 were females (38.8%). The mean age at the time of OSCC diagnosis was 63.9 years, ranging from 23 to 87 years. The distribution of tumors was as follows: 37 patients (75.5%) had tumors in the mobile tongue, 7 (14.3%) in the gingiva, 4 (8.2%) in the floor of the mouth, and 1 (2.0%) in the buccal mucosa. Regarding smoking history, 28 patients (57.1%) reported being current or former smokers, while 21 patients (42.9%) were classified as never smokers. Twenty-one patients (42.9%) had positive pathological nodal status (pN), including micrometastases in two patients. The distribution of pathological T status (pT) was as follows: pT1 – 14 patients (28.6%), pT2 – 17 patients (34.7%), pT3 – 12 patients (24.5%) and pT4 – 6 patients (12.2%). None of the patients had distant metastases, meaning all were staged as M0. Seventeen patients (34.7%) developed recurrence during the follow-up time. A comprehensive overview of the clinicopathological characteristics is provided in **Table 1**, while the complete clinical data for all subjects can be found in **Supplementary Table 1**.

**Table 1:** Demographic characteristics and clinicopathological data of enrolled patients.

Variable	N (%)
Age	
Age <60	16 (32.7)
Age ≥60	33 (67.3)
Sex	
Female	19 (38.8)
Male	30 (61.2)
Smoking history	
Never smoker	21 (42.9)
Previous/current smoker	28 (57.1)
pT status	
pT1	14 (28.6)
pT2	17 (34.7)
pT3	12 (24.5)
pT4	6 (12.2)
pN status	
N0	28 (57.1)
N+	21 (42.9)
Tumour site	
Mobile tongue	37 (75.7)
Gingiva	7 (14.3)
Floor of the mouth	4 (8.2)
Buccal mucosa	1 (2.0)
Recurrence status	
Recurrence-free	32 (65.3)
Recurrence	17 (34.7)

### Expression of PD-1, CTLA-4, TIGIT, TIM-3, LAG-3, CD69 and CXCR5 on CD4+, CD8+ and regulatory T cells in human TDLNs

**Figure 1:**
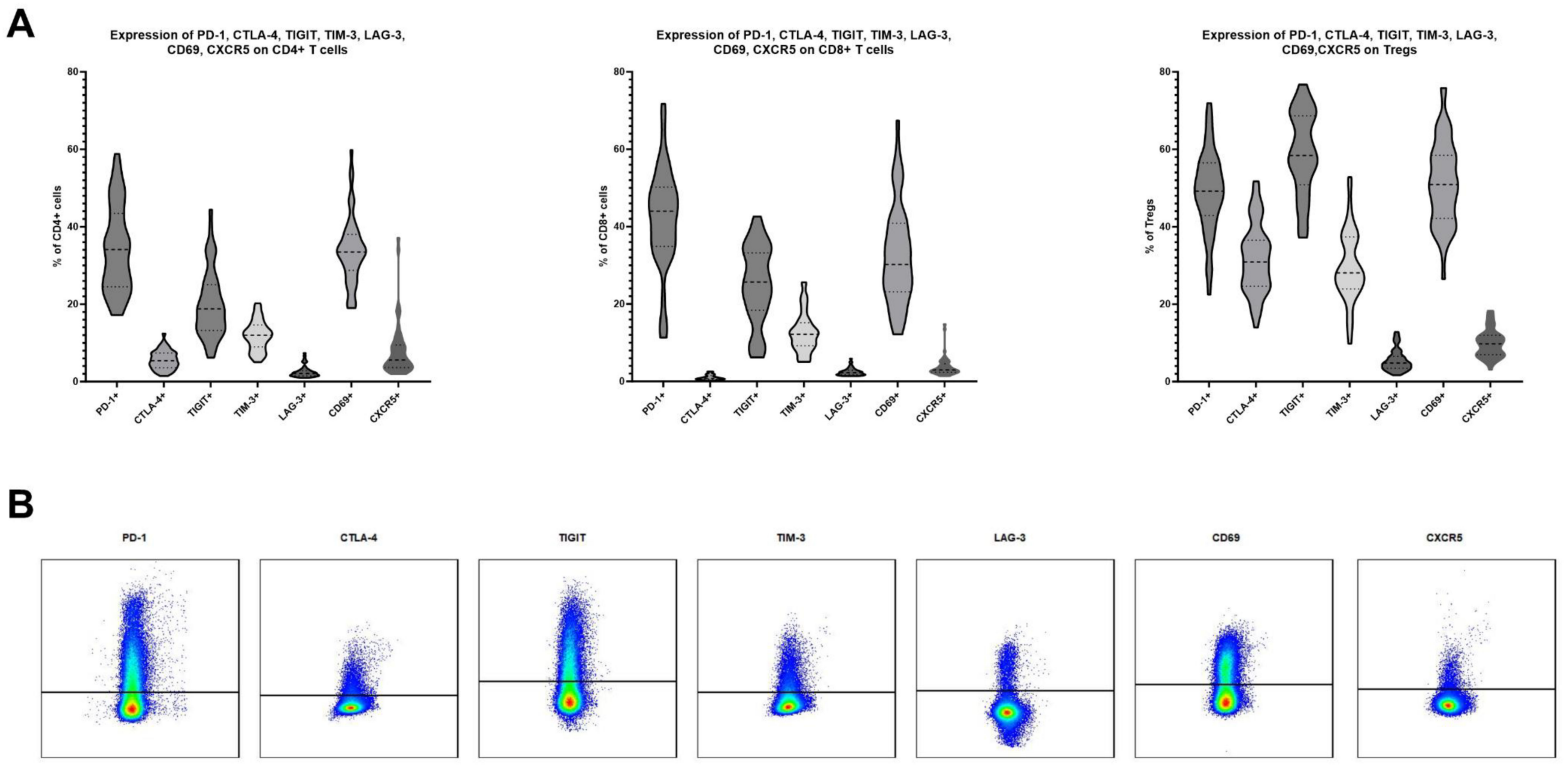
**(A)** Expression levels of PD-1, CTLA-4, TIGIT, TIM-3, LAG-3, CD69 and CXCR5 on CD4, CD8 and T regulatory cells in TDLNs of patients suffering from oral squamous cell carcinoma. 49 samples were analyzed. **(B)** An exemplary gating of positive populations on CD4 cells for investigated markers.

Forty-nine TDLNs were subjected to multicolor flow cytometry analysis, following the protocol outlined in the Materials and Methods section. The average expression levels (mean ± SD) on CD4 T cells were as follows: PD-1, 34.39% ± 11.10; CTLA-4, 5.60% ± 2.40; TIGIT, 20.11% ± 8.65; TIM-3, 11.90% ± 3.83; LAG-3, 2.54% ± 1.46; CD69, 33.94% ± 8.81; and CXCR5, 8.04% ± 7.12 (**Figure 1A**). The average expression levels (mean ± SD) on CD8 T cells were as follows: PD-1, 42.15% ± 13.04; CTLA-4, 1.04% ± 0.64; TIGIT, 25.02% ± 10.07; TIM-3, 12.59% ± 5.10; LAG-3, 2.49% ± 0.98; CD69, 32.72% ± 12.78; and CXCR5, 3.78% ± 2.58 (**Figure 1A**). T regulatory cells defined as CD4+FoxP3+ cells were characterized by higher expression levels of all analyzed markers. The average expression levels (mean ± SD) on Tregs were as follows: PD-1, 49.17% ± 10.56; CTLA-4, 31.78% ± 8.95; TIGIT, 58.88% ± 10.74; TIM-3, 29.71% ± 9.14; LAG-3, 5.47% ± 2.77; CD69, 51.39% ± 10.82; and CXCR5, 9.90% ± 3.59 (**Figure 1A**). Representative flow cytometry plots of the studied markers are shown in **Figure 1B**.

### Correlations between expression of PD-1, CTLA-4, TIGIT, TIM-3 and LAG-3 in TDLNs

**Figure 2:**
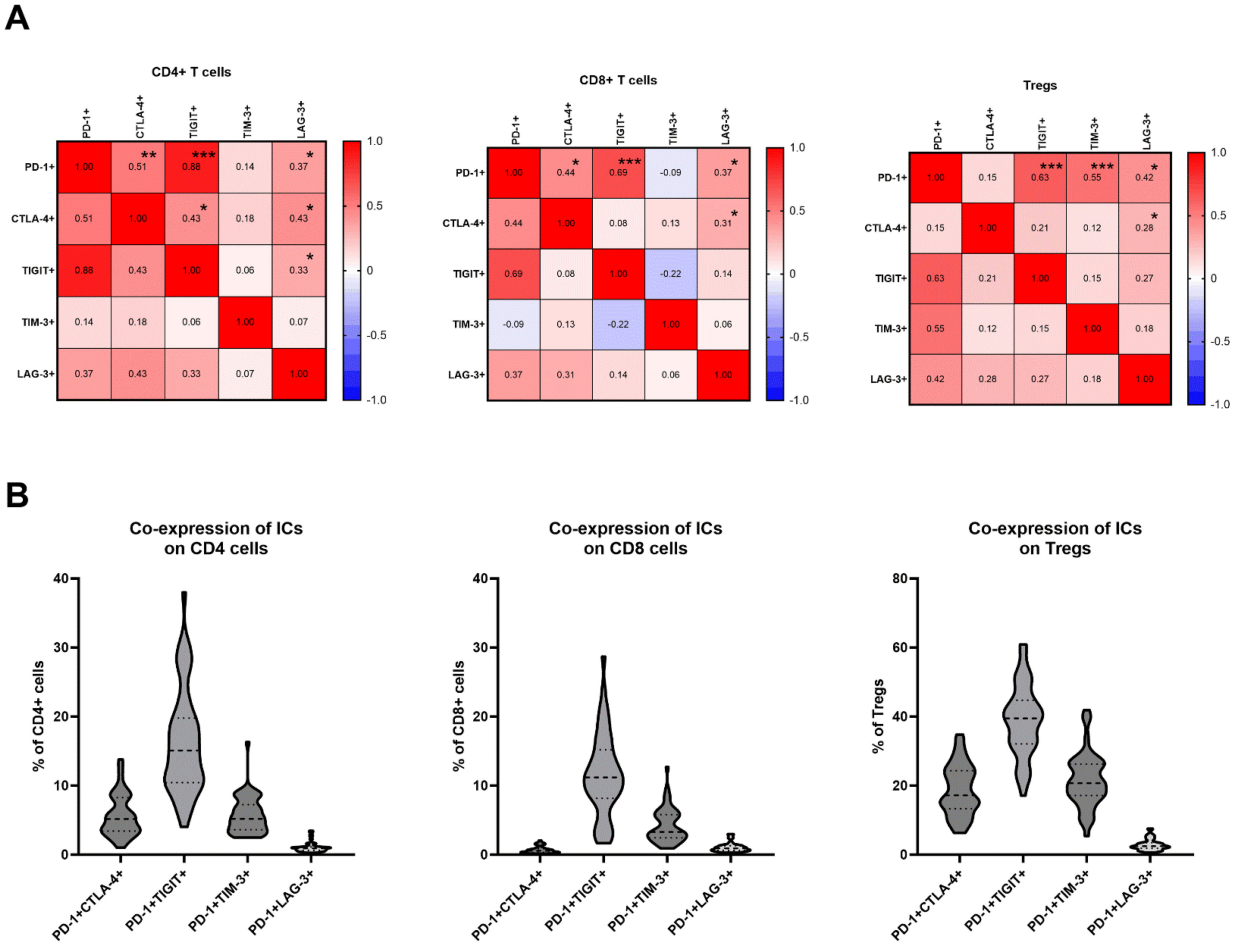
**(A)** Correlation matrix between expression levels of PD-1, CTLA-4, TIGIT, TIM-3 and LAG-3 on CD4, CD8 cells and Tregs; Signifcance is shown by asterisk symbols with significance levels as follows: *p ≤ 0.05, **p<0.001,***p<0.0001. **(B)** Co-expression of PD-1 and other immune checkpoints on CD4, CD8 and T regulatory cells.

The correlation between these markers was explored independently in CD4, CD8, and regulatory T cells using Pearson correlation coefficients. Within CD4 cells, among the 10 pairs investigated, six significant correlations were identified. These correlations varied from weak to strong, with the strongest positive correlation observed between PD-1 and TIGIT positivity (r=0.88, p<0.0001) and PD-1 and CTLA-4 positivity (r=0.51, p=0.0002) (**Figure 2A**). In CD8 cells, four pairs exhibited significant correlations, with the strongest positive correlation found between PD-1 and TIGIT positivity (r=0.69, p<0.0001), as well as PD-1 and CTLA-4 positivity (r=0.44, p=0.0014) (**Figure 2A**). Within T regulatory cells, four pairs demonstrated a significant positive correlation, with the most pronounced associations between PD-1 and TIGIT (r= 0.63, p<0.0001) and PD-1 and TIM-3 (r=0.55, p<0.0001) (**Figure 2A**).

### Co-expression of PD-1 and other immune checkpoint receptors on T cells in TDLNs

The frequencies of T cells co-expressing PD-1 and other immune checkpoint inhibitors were analyzed. The frequencies of CD4+ cells with double positivity were as follows: PD-1+CTLA-4 + 5.64% ± 3.0; PD-1+TIGIT+ 16.36% ± 7.54; PD-1+TIM-3 + 5.75% ± 2.63; PD-1+LAG-3 + 1.08% ± 0.72 (**Figure 2B**). The frequencies of CD8+ cells with double positivity were as follows: PD-1+CTLA-4 + 0.70% ± 0.49; PD-1+TIGIT+ 11.56% ± 5.90; PD-1+TIM-3 + 4.24% ± 2.46; PD-1+LAG-3 + 1.08% ± 0.66 (**Figure 2B**). The frequencies of Tregs with double positivity were as follows: PD-1+CTLA-4 + 18.77% ± 7.35; PD-1+TIGIT+ 38.86% ± 10.21; PD-1+TIM-3 + 21.54% ± 7.74; PD-1+LAG-3 + 2.90% ± 1.62 (**Figure 2B**).

### Expression of immune checkpoints and frequency of Tregs in TDLNs is associated with recurrence status

**Figure 3:**
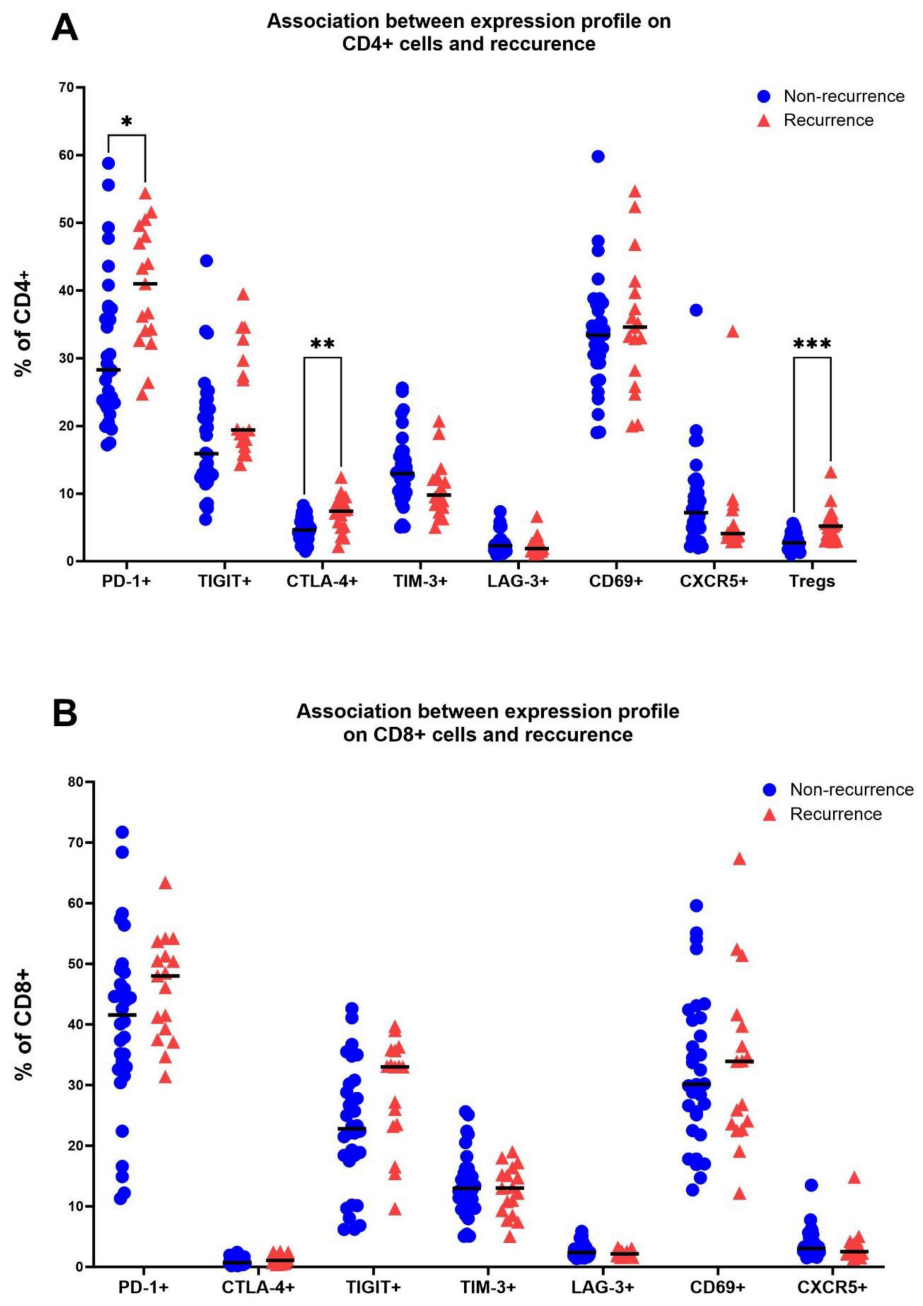
Association between the recurrence status (no recurrence/recurrence) and levels of PD-1, CTLA-4, TIGIT, TIM-3, LAG-3, CD69 and CXCR5 on CD4 cells in TDLNs **(A)** and on CD8 cells in TDLNs **(B)**. Multiple t-test was performed and corrected using Holm-Sidak method. Significance is shown by asterisk symbols with significance levels as follows: *p ≤ 0.05, **p<0.001,***p<0.0001.

To reduce Type I error, we used a multiple t-test with *post-hoc* Holm-Sidak method to compare levels of immune checkpoints and other markers between different subgroups. The most notable difference was observed in the levels of FoxP3+ cells in TDLNs between patients with and without recurrence. In the former group, the mean frequency of FoxP3+ cells was 5.53% ± 2.6, while in the latter group, it was 2.96% ± 1.31 (adjusted p=0.0002) (**Figure 3A**). Additionally, significantly elevated levels of CD4+PD-1+ (adjusted p=0.0275), and CD4+CTLA-4+ (adjusted p=0.0064) were observed in the cohort that developed recurrence (**Figure 3A**). Patients with recurrence also exhibited a trend for a higher expression of TIGIT on CD4+ cells and lower levels of CD4+CXCR5+ cells in TDLNs compared to those without recurrence (**Figure 3A**). However, these associations were not significant when corrected for multiple comparisons with Holm-Sidak method. Within CD8 T cells, there were no significant differences between cohorts when using the multiple t-test method. There was a trend for higher levels of TIGIT+ expression in the recurrence group (**Figure 3B**).

### Association of immune checkpoint receptors in TDLNs with metastasis status, T- and N- stage

We further compared the expression levels of PD-1, CTLA-4, TIGIT, TIM-3, LAG-3, CD69, and CXCR5 between following subgroups: non-metastatic TDLN vs metastatic TDLN, pT1-T2 vs pT3-T4 tumors and pN0 vs pN+ patients. The only significant difference was observed for expression of CTLA-4 on CD4 cells in relation to the size of the tumour. Patients with pT1-T2 tumours had significantly lower levels of CTLA-4 on CD4 cells when compared to patients with pT3-T4 lesions (adjusted p= 0.0181). For remaining markers and subgroups no other significant associations were detected when using multiple t test method with *post-hoc* Holm-Sidak. Please see **Supplementary Figure 1** for details.

### Expression levels of immune checkpoint receptors on T cells in TDLNs strongly correlate with disease-free survival

**Figure 4:**
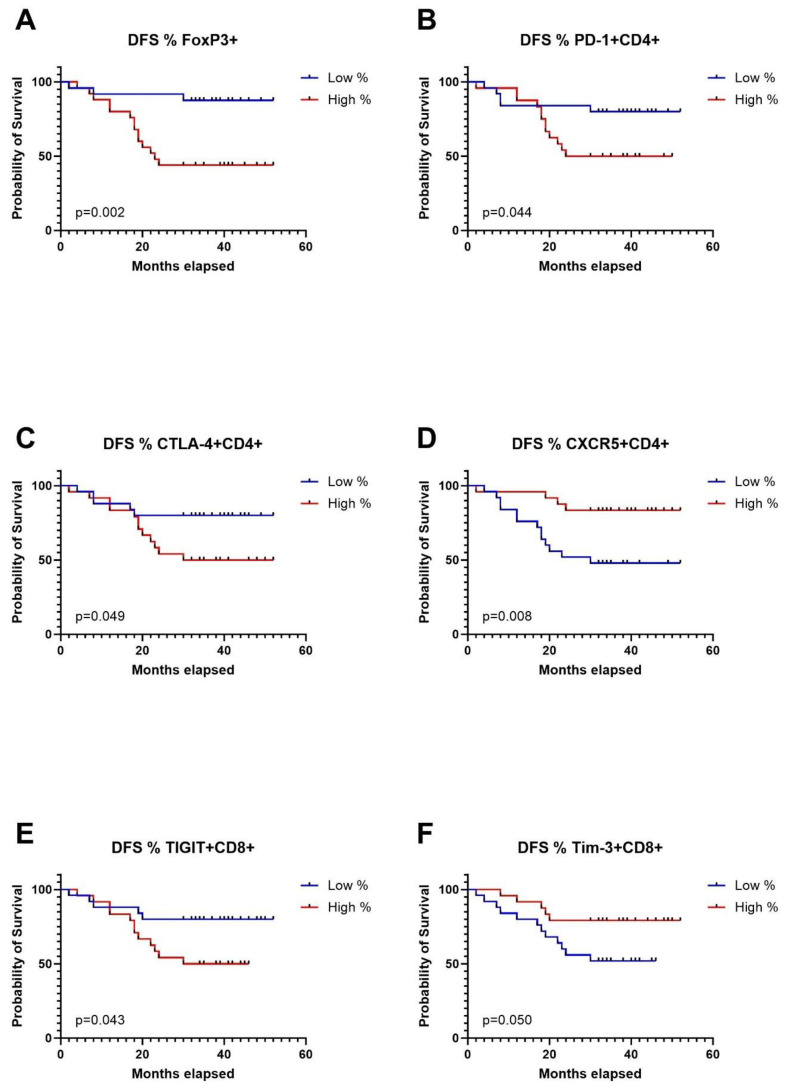
Kaplan-Meier curves for disease free survival (DFS) in relation to levels of Tregs in TDLNs **(A)**, PD-1 expression **(B)**, CTLA-4 expression **(C)**, CXCR5 expression **(D)** on CD4 cells in TDLNs and TIGIT expression **(E)** and TIM-3 expression **(F)** on CD8 cells in TDLNs.

**Figure 5:**
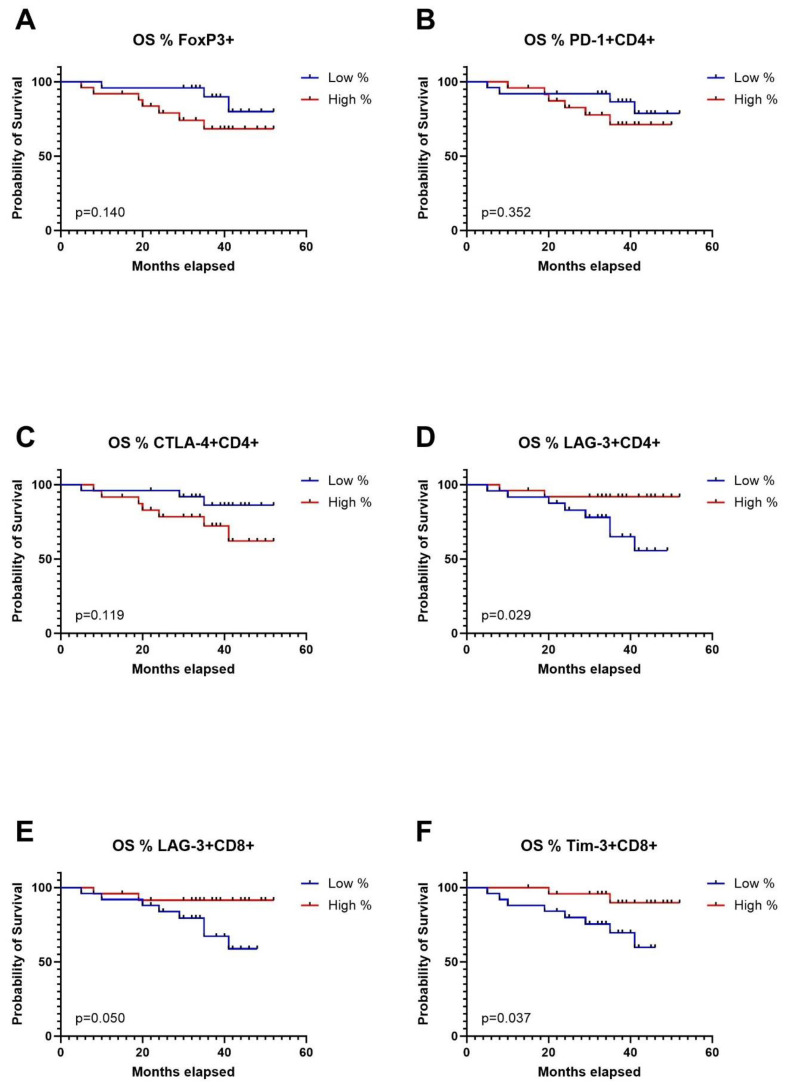
Kaplan-Meier curves for overall survival (OS) in relation to levels of Tregs in TDLNs **(A)**, PD-1 expression **(B)**, CTLA-4 expression **(C)**, LAG-3 expression **(D)** on CD4 cells in TDLNs and LAG-3, TIM-3 expression **(E)** and TIM-3 expression **(F)** on CD8 cells in TDLNs.

To conduct survival analysis, we categorized the expression of markers into low and high groups, using the median as the threshold. In the Kaplan-Meier analysis, patients in the high group for FoxP3+, CD4+PD1+, CD4+CTLA-4+, and CD8+TIGIT+ exhibited significantly shorter disease-free survival (DFS). Conversely, patients in the high group for CD4+CXCR5+ and CD8+TIM-3 showed significantly longer DFS (**Figures 4A–F**). Regarding overall survival (OS), patients in the low group for CD4+LAG-3+, CD8+LAG-3+, and CD8+TIM-3+ demonstrated significantly worse outcomes compared to patients with high expression of aforementioned markers in TDLNs (**Figures 5A–F**). A summary of p-values, 3-year DFS, and OS is presented in **Table 2**.

**Table 2:** Kaplan Meier analysis for 3 years DFS and OS in relation to expression of FoxP3+, PD-1, CTLA-4, TIGIT, TIM-3, LAG-3, CD69 and CXCR5 on CD4 and CD8 cells in TDLNs.

Variable	Level	DFS		OS	
		3 years DFS	p-value (log-rank test)	3 years OS	p-value (log-rank test)
FoxP3+	Low	87.5%	**0.0018**	89.8%	0.1397
	High	44.0%		68.4%	
CD4+PD-1+	Low	80.0%	**0.0438**	86.5%	0.3521
	High	50.0%		71.2%	
CD4+CTLA4+	Low	80.0%	**0.0490**	86.1%	0.1190
	High	50.0%		72.3%	
CD4+TIGIT+	Low	70.8%	0.5761	77.8%	0.4827
	High	60.0%		80.7%	
CD4+TIM-3+	Low	72.0%	0.2888	79.6%	0.5321
	High	58.3%		78.3%	
CD4+LAG-3+	Low	54.2%	0.1083	65.0%	**0.0293**
	High	76.0%		91.8%	
CD4+CXCR5+	Low	48.0%	**0.0080**	70.2%	0.1356
	High	83.3%		87.5%	
CD4+CD69+	Low	73.9%	0.3727	84.8%	0.8261
	High	60.0%		77.2%	
CD8+PD-1+	Low	72.0%	0.2750	78.6%	0.8223
	High	58.3%		80.6%	
CD8+CTLA4+	Low	72.0%	0.3188	82.0%	0.3965
	High	58.3%		76.6%	
CD8+TIGIT+	Low	80.0%	**0.0426**	80.0%	0.7977
	High	50.0%		74.2%	
CD8+TIM-3+	Low	52.0%	**0.0499**	69.6%	0.370
	High	79.2%		89.7%	
CD8+LAG-3+	Low	56.0%	0.2009	67.2%	0.0503
	High	75.0%		91.5%	
CD8+CXCR5+	Low	61.5%	0.5606	77.2%	0.5662
	High	69.5%		81.2%	
CD8+CD69+	Low	68.0%	0.7340	74.3%	0.4944
	High	62.5%		84.4%	

bold values - statistically significant p value.

### Univariate and multivariate analyses for disease free survival and overall survival

According to the Cox proportional-hazards models for disease-free survival (DFS), the univariate analysis indicated associations between T status, smoking history, levels of FoxP3+ cells, CD4+PD-1+ cells, CD4+CTLA-4+ cells, CD4+CXCR5+ cells, and CD8+TIGIT+ cells in TDLNs and the prognosis of patients with OSCC. However, the multivariate analysis revealed that only the levels of FoxP3+ cells, CD4+CXCR5+ cells, and CD8+TIGIT+ cells in TDLNs were independent predictors of DFS in this cohort (see **Table 3**). As shown in the table, patients exhibiting elevated levels of FoxP3+CD4+ cells and CD8+TIGIT+ in their TDLNs were associated with a higher risk of recurrence (HR 7.196, 95% CI 1.656-31.271, p=0.008 and HR 3.413, 95% CI 1.025-11.366, p=0.045, respectively). Conversely, those with high levels of CD4+CXCR5+ cells in TDLNs demonstrated a significantly lower risk for recurrence (HR 0.267, 95% CI 0.078-0.911, p=0.035).

**Table 3:** Univariate and multivariate Cox proportional hazards regression model for disease free survival (DFS) and overall survival (OS).

Parameter	Variable	DFS				OS			
		Univariate analysis Hazard ratio (95%CI)	P value	Multivariate analysis Hazard ratio (95%CI)	P value	Univariate analysis Hazard ratio (95%CI)	P value	Multivariate analysis Hazard ratio (95%CI)	P value
T-stage	T1-T2	Ref.	**0.002**	Ref.	0.139	Ref.	**0.003**	Ref.	0.049
	T3-4	7.944 (2.094-30.135)		2.300 (0.763-6.936)		13.333 (2.389-74.404)		5.306 (1.009-27.917)	
N-stage	N0	Ref.	0.301			Ref.	**0.016**	Ref.	0.077
	N+	1.875 (0.570-6.171)				8.000 (1.481-43.202)		4.851 (0.843-27.912)	
Smoking	Never	Ref.	**0.034**	Ref.	0.446	Ref.	**0.033**	Ref.	0.059
	Previous/ Current	4.179 (1.116-15.650)		1.690 (0.438-6.522)		10.500 (1.211-91.026)		9.024 (0.919-88.622)	
%FoxP3+ of CD4	Low	Ref.	**0.003**	Ref.	**0.008**	Ref.	0.188		
	High	8.909 (2.101 – 37.778)		7.196 (1.656-31-271)		2.722 (0.612-12.101)			
%PD-1 of CD4	Low	Ref.	**0.032**	Ref.	0.686	Ref.	0.438		
	High	4.000 (1.129-14.175)		0.750 (0.186-3.021)		1.750 (0.426-7.190)			
%CTLA-4 of CD4	Low	Ref.	**0.032**	Ref.	0.993	Ref.	0.147		
	High	4.000 (1.129-14.175)		1.006 (0.292-3.464(		3.020 (0.678-13.422)			
%CXCR5 of CD4	Low	Ref.	**0.013**	Ref.	**0.035**	Ref.	0.188		
	High	0.185 (0.049-0.698)		0.267 (0.078-0.911)		0.367 (0.83-1.633)			
%TIGIT of CD8	Low	Ref.	**0.032**	Ref.	**0.045**	Ref.	0.942		
	High	4.000 (1.129-14.175)		3.413 (1.025-11.366)		1.053 (0.262-4.224)			

bold values - statistically significant p value.

Through the Cox proportional-hazards models for OS, the univariate analysis indicated significant associations between T status, N status and smoking history and prognosis. In the multivariate analysis, the factors that reached significance in the univariate analysis were included, but only T status remained an independent predictor of overall survival (see **Table 3**).

## Discussion

In this study, we have explored the expression of immune checkpoints on T cells in TDLNs in patients with OSCC. The findings reveal varying levels of expression, with PD-1 and TIGIT showing high levels, CTLA-4 and TIM-3 exhibiting moderate levels, and LAG-3 demonstrating low expression on T cells in TDLNs. Furthermore, the study demonstrated the prognostic significance of immune checkpoints expression in TDLNs for DFS and OS. Elevated levels of FoxP3+CD4+ cells and CD8+TIGIT+ cells correlated with significantly worse DFS in multivariate model, while high CD4+CXCR5+ levels were associated with better DFS. T status emerged as an independent predictor of OS.

Accumulating evidence indicates a substantial impact of the immune response status on cancer progression and metastasis. It is widely acknowledged that the abnormal immunosurveillance and immune escape mechanisms employed by tumor cells are pivotal factors shaping the antitumor immune response, ultimately contributing to cancer progression and mortality (16). Proof of the significance of a well-functioning anti-cancer immune system is the demonstrated efficacy of immunotherapy, with anti-PD-1 and anti-CTLA-4 antibodies standing out as notable examples. Immunotherapy in an adjuvant setting, after primary treatment including removal of TDLNs, is characterized by low response rates (<20%) in patients with HNSCC (17, 18). One plausible explanation for these low response rates is the absence of TDLNs, which have been previously removed as a part of standard of care in HNSCC. In contrast, employing immunotherapy in the neoadjuvant setting, while TDLNs are still in place, yields more promising outcomes, with response rates ranging from 17-50% (9–11). In this study, we provide a further rationale for neoadjuvant use of immune checkpoint inhibitors, as we proved that PD-1, TIGIT, CTLA-4 and TIM-3 were abundantly expressed on T cells in TDLNs and patients with highest levels of immune checkpoints in TDLNs had the highest risk for recurrence regardless of TNM staging. Thus, one can hypothesize that these patients would benefit the most from neoadjuvant immunotherapy.

The explanation to why patients with high levels of CD4+PD-1+, CD4+TIGIT+, CD4+CTLA-4+, CD8+TIGIT+ cells in their TDLNs have a higher risk for recurrence could be that the expression of immune checkpoints can represent the exhaustion-state of the immune system or the ability of the tumor cells to escape the anti-cancer immunity. The presence of high levels of immunosuppressive T regulatory cells and low levels of CXCR5+ cells in TDLNs of patients who relapse further reinforces this hypothesis, reflecting the weakness of the immune system and tumour-induced immunosuppressive environment in TDLNs. The levels of expression of the investigated immune checkpoints in our cohort were in line with the findings of Seifert et al. who performed flow cytometric analysis of TDLNs in pancreatic cancer. They showed a high expression of PD-1 and CTLA-4 in TDLNs, moderate expression of TIM-3 and low expression of LAG-3 (19). The most notable disparity between our study and that of Seifert et al. was that in our samples CD8+ cells expressed lower levels of CTLA-4 as compared to TLDNs in pancreatic cancer. The association of high levels of PD-1 on T cells in TDLNs with recurrence is in line with our previous study on a smaller cohort, where we showed that patients with high levels of PD-1 positive CD3+ cells had significantly worse short-term DFS and OS (20). Furthermore, Tregs populations in TDLNs were previously the subject of investigation in our previous study in OSCC (21) and in different cancer types such as colorectal cancer, lung cancer, gastric cancer and several other cancer types (22–24). The aforementioned studies showed that there is an association between high levels of Tregs in TDLNs with more advanced disease stages, suggesting an immunosuppressive environment in TDLNs. Our group also previously characterized B regulatory cells in TDLNs and showed that there was significantly higher accumulation of Bregs in TDLNs as compared to non-TDLNs, indicating immunosuppressive environment in TDLNs (25).

Furthermore, our results revealed that a significant fraction of T cells within TDLNs co-express several immune checkpoints inhibitors. We detected high levels of PD-1+ and TIGIT+ co-expressing cells in TDLNs, with the highest fraction within T regulatory cells. PD-1+CTLA-4 and PD-1+TIM-3+ cells were also present at moderate levels within the analyzed nodes. Thommen et al. showed previously that co-expression of several immune checkpoints on tumour infiltrating lymphocytes (TILs) was associated with a severely exhausted T cell state (26). Several other studies showed that increased co-expression of immune checkpoint (IC) on TILs is associated with poorer prognosis (27, 28). However, the co-expression of ICs on T cells in TDLNs in OSCC was not previously investigated and here we showed that this phenomenon is also present in this anatomical compartment. Further investigation is needed to determine the clinical significance of co-expressing cells in TDLNs. However, the observed co-expression gives further rationale for using a combination of immune checkpoint inhibitors such as anti-PD-1/anti-TIGIT or anti-PD-1/anti-CTLA-4 in the neoadjuvant setting for treatment of HNSCC.

Our results suggest that the immunological traits observed in TDLNs serve as robust indicators of survival, especially in terms of disease-free survival. This information can be a valuable enhancement for clinicians when drawing follow-up plans, and may even play a pivotal role in deciding whether to implement adjuvant therapies, such as neck radiotherapy, in patients deemed at high risk. However, it is crucial to recognize certain limitations in our study, with the cohort size and follow-up duration being the most significant factors limiting our findings. Further investigations with larger cohorts and longitudinal studies are warranted to validate these findings and explore potential therapeutic interventions based on immune checkpoint modulation in OSCC. Furthermore, in this cohort we could only present 3 years survival and it is possible that the result, especially for OS will be different at the 5-years’ time point.

## Conclusions

In summary, this paper demonstrates a significant association between the immune signatures of TDLNs and disease progression in OSCC. It shows that a higher frequency of FoxP3+ regulatory T cells and CD8+ cells expressing TIGIT within TDLNs is indicative of a reduced DFS, while an abundance of CD4+ cells expressing CXCR5 is predictive of a more favorable DFS outcome. The prognostic significance of immune signatures in TDLN is a compelling argument for the use of immunotherapy in the neoadjuvant setting. By intervening at a stage when the immune systems responsiveness may be more robust, there is the potential to disrupt the disease trajectory, thereby reducing the likelihood of recurrence and improving overall survival.

## Data Availability

The raw data supporting the conclusions of this article will be made available by the authors, without undue reservation.

## Supplementary material

Supplementary Figure 1 Association between metastatic vs non-metastatic TDLNs **(A)**, T1-T2 vs T3-T4 stage **(B)**, N0 vs N+ stage **(C)** and levels of PD-1, CTLA-4, TIGIT, TIM-3, LAG-3, CD69 and CXCR5 on CD4 cells and on CD8 cells in TDLNs Multiple t-test was performed and corrected using Holm-Sidak method. Significance is shown by asterisk symbols with significance levels as follows: *p ≤ 0.05, **p<0.001,***p<0.0001.
