# Supplementary figures and images for: Prognostic value of T regulatory cells and immune checkpoints expression in tumor-draining lymph nodes for oral squamous cell carcinoma
Source: Front Immunol. 2024 Oct 15;15:1455426. doi: 10.3389/fimmu.2024.1455426 (PMC11518749; doi:10.3389/fimmu.2024.1455426)

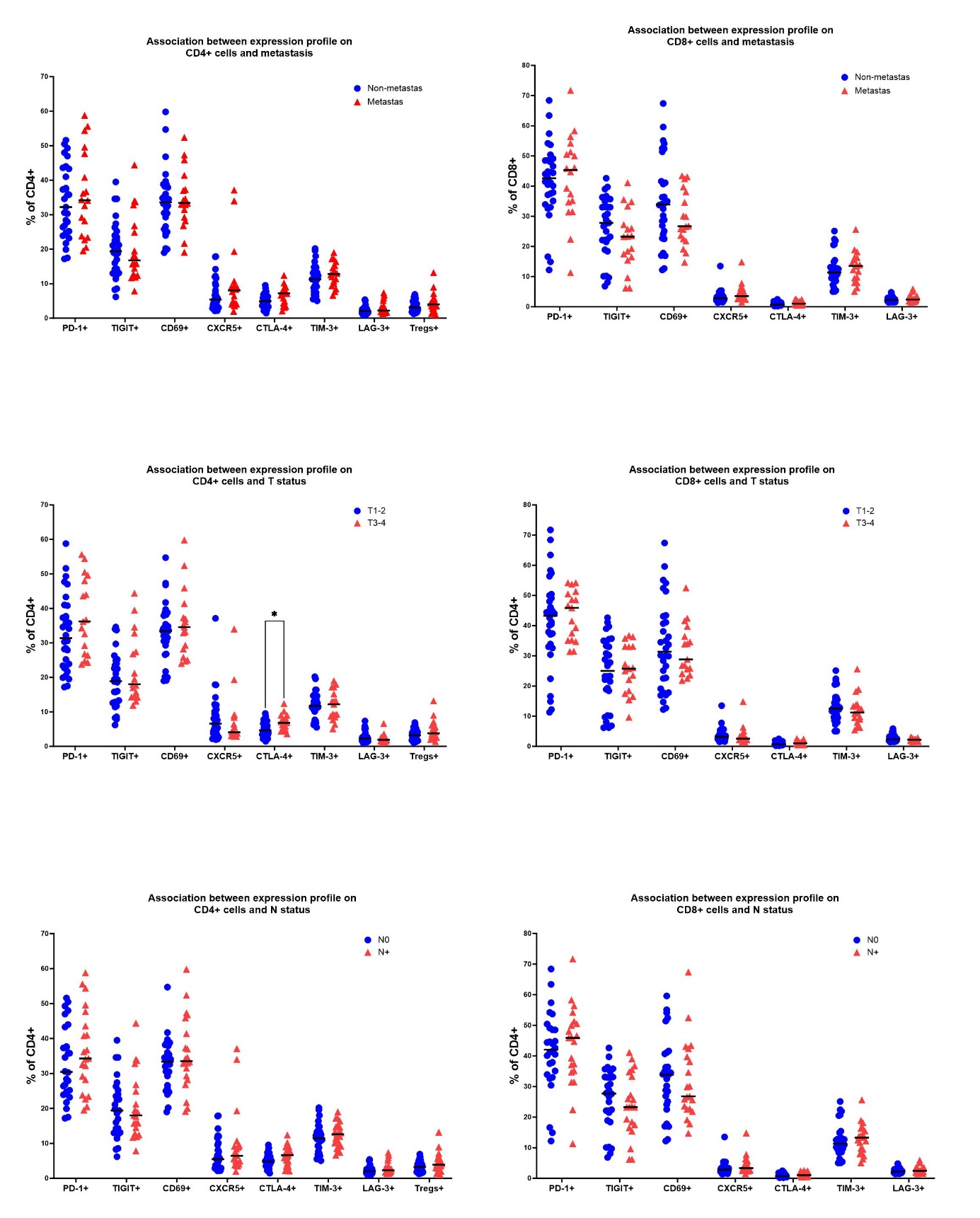


Supplementary Figure 1.

Supplement: Supplementary Figure 1 — Association between metastatic vs non-metastatic TDLNs (A), T1-T2 vs T3-T4 stage (B), N0 vs N+ stage (C) and levels of PD-1, CTLA-4, TIGIT, TIM-3, LAG-3, CD69 and CXCR5 on CD4 cells and on CD8 cells in TDLNs Multiple t-test was performed and corrected using Holm-Sidak method. Significance is shown by asterisk symbols with significance levels as follows: *p ≤ 0.05, **p<0.001,***p<0.0001. [file DataSheet1.docx]
