# Supplementary material for: Prognostic value of T regulatory cells and immune checkpoints expression in tumor-draining lymph nodes for oral squamous cell carcinoma
Source: Front Immunol. 2024 Oct 15;15:1455426. doi: 10.3389/fimmu.2024.1455426 (PMC11518749; doi:10.3389/fimmu.2024.1455426)
Supplement: Supplementary file 2 [file Table1.docx]

Supplementary Table 1.

| **ID** | **Age** | **Sex** | **Localization** | **Smoking** | **pT** | **pN** | **Region of the neck** | **Metastatic TDLN (Yes/No)** | **Time to Relapse (months)** | **Time to Death (months)** |
| --- | --- | --- | --- | --- | --- | --- | --- | --- | --- | --- |
| 1 | 70 | F | T | Yes | T3 | N2b | 2 | Yes | 19 | 20 |
| 2 | 73 | M | T | No | T2 | N2c | 3 | No | 18 | X |
| 3 | 43 | F | T | Yes | T2 | N0 | 2 | No | 24 | X |
| 4 | 75 | F | T | Yes | T3 | N0 | 1 | No | 30 | 41 |
| 5 | 51 | M | T | Yes | T3 | N2b | 2 | Yes | 7 | X |
| 6 | 65 | M | F | Yes | T4a | N3b | 3 | Yes | 20 | 35 |
| 7 | 72 | M | T | Yes | T3 | N2c | 3 | Yes | 22 | 24 |
| 8 | 75 | F | T | Yes | T3 | N0 | 2 | No | 17 | 29 |
| 9 | 78 | M | T | Yes | T3 | N3b | 2 | Yes | 19 | 19 |
| 10 | 71 | M | G | Yes | T4a | N0 | 3 | No | 8 | X |
| 11 | 64 | F | G | Yes | T4a | N2b | 2 | Yes | 2 | 10 |
| 12 | 56 | F | T | Yes | T2 | N1 | 3 | Yes | 4 | 5 |
| 13 | 76 | M | F | Yes | T1 | N0 | 1 | No | 12 | X |
| 14 | 78 | F | T | No | T1 | N1 | 1 | Yes | 28 | 35 |
| 15 | 87 | M | G | Yes | T4a | N0 | 2 | No | 12 | X |
| 16 | 67 | M | T | No | T1 | N0 | 2 | No | 18 | X |
| 17 | 58 | F | G | Yes | T4a | N0 | 2 | No | 23 | X |
| 18 | 32 | F | T | Yes | T1 | N0 | 2 | No | X | X |
| 19 | 80 | M | G | No | T2 | N0 | 1 | No | X | X |
| 20 | 74 | M | T | No | T1 | N0 | 2 | No | X | X |
| 21 | 44 | F | T | No | T1 | N0 | 2 | No | X | X |
| 22 | 78 | F | G | No | T1 | N0 | 1 | No | X | X |
| 23 | 70 | M | F | Yes | T1 | N0 | 2 | No | X | X |
| 24 | 23 | M | T | No | T2 | N0 | 2 | No | X | X |
| 25 | 46 | F | T | No | T3 | N0 | 2 | No | X | X |
| 26 | 64 | F | T | Yes | T2 | N0 | 1 | No | X | X |
| 27 | 70 | M | T | No | T1 | N0 | 2 | No | X | X |
| 28 | 51 | M | T | Yes | T2 | N0 | 1 | No | X | X |
| 29 | 60 | M | T | Yes | T1 | N0 | 2 | No | X | X |
| 30 | 78 | F | T | No | T2 | N0 | 2 | No | X | X |
| 31 | 64 | M | T | Yes | T2 | N0 | 3 | No | X | X |
| 32 | 56 | F | T | Yes | T1 | N0 | 2 | No | X | X |
| 33 | 57 | M | T | No | T3 | N0 | 2 | No | X | X |
| 34 | 70 | F | T | No | T2 | N0 | 2 | No | X | X |
| 35 | 68 | M | T | Yes | T2 | N0 | 2 | No | X | X |
| 36 | 55 | M | B | Yes | T2 | N0 | 1 | No | X | X |
| 37 | 62 | M | T | No | T1 | N0 | 2 | No | X | X |
| 38 | 54 | F | T | No | T1 | N2b | 1 | Yes | X | X |
| 39 | 69 | M | G | Yes | T4a | N1 | 3 | Yes | X | X |
| 40 | 67 | M | T | No | T1 | N1 | 2 | Yes | X | X |
| 41 | 82 | F | F | No | T2 | N0(i+) | 1 | Yes | X | X |
| 42 | 70 | M | T | No | T2 | N1 | 5 | Yes | X | X |
| 43 | 47 | M | T | No | T2 | N1 | 2 | Yes | X | X |
| 44 | 50 | F | T | No | T3 | N2b | 1 | Yes | X | X |
| 45 | 72 | M | T | Yes | T2 | N0(i+) | 3 | Yes | X | X |
| 46 | 72 | M | T | Yes | T3 | N1 | 2 | Yes | X | X |
| 47 | 69 | M | T | Yes | T2 | N2b | 2 | Yes | X | X |
| 48 | 63 | M | T | Yes | T3 | N1 | 2 | No | X | X |
| 49 | 55 | M | T | No | T3 | N3b | 2 | Yes | X | X |

Abbreviations: M – male; F- female; T – mobile tongue, G – gingiva, F – floor of the mouth, B – buccal mucosa
